# Sustainable Elimination of Schistosomiasis in Ethiopia—A Five-Year Follow-Up Study

**DOI:** 10.3390/tropicalmed7090218

**Published:** 2022-09-01

**Authors:** Lotte Ben Gal, Michal Bruck, Robyn Tal, Sarit Baum, Jemal Mahdi Ali, Lemlem Legesse Weldegabriel, Galia Sabar, Rachel Golan, Zvi Bentwich

**Affiliations:** 1 NALA, Carlebach 29, Tel Aviv-Yafo 6713224, Israel; 2 Department of Microbiology, Immunology and Parasitology, College of Medicine and Health Sciences, University of Gondar, Gondar P.O. Box 196, Ethiopia; 3 Tigray Regional Health Bureau, Tigray P.O. Box 07, Ethiopia; 4 The Department of Middle Eastern and African History, Tel Aviv University, Tel Aviv-Yafo P.O. Box 39040, Israel; 5 Department of Epidemiology, Biostatistics and Community Health Sciences, Ben-Gurion University of the Negev, Beer Sheva P.O. Box 653, Israel; 6 Shraga Segal Department of Microbiology, Immunology and Genetics, Ben-Gurion University of the Negev, Beer Sheva P.O. Box 653, Israel

**Keywords:** schistosomiasis, *S. mansoni*, endemic region, control, Ethiopia, behavioral change, NALA

## Abstract

In 2009, Mekele, the capital of the Tigray Region in Ethiopia, presented a mean prevalence of 44.7% of schistosomiasis (*S. mansoni*) in school children. Termed a public health problem, NALA, an international public health non-governmental organization, and their partners implemented a novel model of intervention, which aimed to compliment mass drug administration (MDA) campaigns with behavioral change (BC) and improved sanitation to achieve sustained elimination of schistosomiasis. The four-year intervention (2009–2012) covered 38 primary schools. The objective of this study was to examine factors associated with control or resurgence of the disease, and the association between the behavioral change program and disease prevalence, ten years after initiation. Eleven primary schools were selected for this follow-up study. All students provided a stool sample and filled in a knowledge, attitude and practice (KAP) questionnaire. In seven out of eleven schools (63.6%) the prevalence of schistosomiasis was maintained below 2% ten years after the initiation of the intervention. In four schools, prevalence returned to pre-intervention levels, defining them as persistent hot spots (PHS). Students from PHS schools scored lower on KAP questionnaires compared to students from responder schools; 3.9 ± 0.9 vs. 4.2 ± 0.9 (*p*-value < 0.001) for practice questions and 4.4 ± 1.4 vs. 4.6 ± 1.5 (*p*-value = 0.03) for attitude questions. The prevalence of schistosomiasis correlated positively with age, (*p*-value = 0.049), sex (relative risk = 1.7, *p*-value < 0.001), and location. Semi-urban locations (*n* = 382) had higher disease prevalence than urban locations (*n* = 242), (22.7% vs. 5.5%, *p*-value < 0.001). Students residing in semi-urban areas and close to a river (<500 m) were at higher risk of contracting schistosomiasis than those living in urban areas far from the river (RR = 5.95, *p*-value < 0.001). Finally, a correlation between prevalence and proximity of schools to rivers was found (semi-urban areas; RR = −0.91, *p*-value = 0.001 vs. urban areas; RR = −0.51, *p*-value = 0.001). Soil-transmitted-helminths prevalence in 2009 was 8.1% and declined during the intervention years to 0.5%. Prevalence in 2018 was found to be stable at 0.8%. These results demonstrate the long-term success of NALAs’ comprehensive model of intervention for elimination of schistosomiasis in school children, combining behavioral change and improved sanitation with MDA.

## 1. Introduction

Schistosomiasis, a snail-transmitted trematode infection, is estimated to currently affect over 240 million people globally, with 90% of the disease burden found in Sub-Saharan Africa [1]. People become infected when the larval forms of the parasite, released by freshwater snails, penetrate the skin during contact with infested freshwater, resulting in the long-term morbidity and, in severe cases, death. Largely endemic in rural areas, impoverished and marginalized communities disproportionately suffer from the disease, as it is associated with a lack of access to adequate sanitation and safe water [2,3].

Praziquantel (PZQ) is highly effective in treating mature schistosomes and is hence distributed as preventive chemotherapy to school aged children, the age group with the highest infection burden [4,5]. As a result of drug effectivity, the Ethiopian Federal Ministry of Health (FMoH) established a national mass drug administration (MDA) program for schistosomiasis management [6]. However, despite the ability of MDA campaigns to reduce schistosomiasis prevalence, reinfection, poor compliance and inefficacy of PZQ in treating juvenile schistosomes have only resulted in short-term successes [7,8,9,10,11]. Moreover, in endemic areas, continued transmission occurs as a result of limited access to safe water, and lack of healthy behaviors habits [12,13,14]. Therefore, integrated approaches combining MDAs with water, sanitation and hygiene (WASH) improvements and behavioral change communication (BCC) that encourage healthy behavior practices, are necessary to interrupt transmission and achieve sustained elimination of the disease [15,16].

In 2009, a deworming project was launched in Mekele, the capital city of Tigray, the most northern Ethiopian region, through a collaboration between the Tigray Regional Health Bureau (TRHB), Organization of Social Services Health and Development (OSSHD) and NALA, an international public health non-governmental organization [17]. Since former evidence has shown that an MDA alone is insufficient for achieving sustained control and elimination of schistosomiasis and soil-transmitted helminthiasis (STH) [7,9], a novel model was implemented. This comprehensive and holistic model combined an MDA with intensive health education promoting BCC practices alongside WASH improvements. The educational curriculum focused on promoting healthy behaviors, such as encouraging clean bodies, clean water and clean environments, and decreasing harmful practices, such as open defecation and swimming in infected rivers. A total of 1871 students from 38 schools participated in the stool survey. Prior to the baseline survey and throughout the intervention, training on prevention of NTDs, particularly on prevention of helminth infections (STH infection and schistosomiasis) was given to health care workers, school teachers and community volunteers. Health education materials on prevention of helminth infections were printed in the local language (“Tigrigna”) and distributed to all the schools after the baseline survey. In addition, thirty pit latrines were constructed and water taps for hand washing were installed in 30 school compounds. An MDA campaign was conducted biannually in all participating schools. A total of 23,214 (51.2%) of the estimated 45,307 students in Mekele, between the ages of 5–15 years old, were treated. The implementation included collaboration with community workers, local college students, volunteers and the Health Development Army—a volunteer arm of the health sector.

The impact was periodically evaluated from its launch in 2009 until its termination in 2013. A consistent decline in the prevalence of both schistosomiasis (*S. mansoni*) and STH infection were recorded, showing a reduction in the prevalence of schistosomiasis from a mean of 44.7% at baseline, to 12.3% in 2010 and 2.0% in 2013. Similarly, the prevalence of STH reduced from 8.1% at baseline, to 4.4% in 2010 and 0.5% in 2013. National surveys conducted by government representatives presented a further reduction in the prevalence of schistosomiasis among children in Mekele (1.45% in 2014 and 0.4% in 2015), and also for the prevalence of STH (0.7% in 2014 and 0.4% in 2015) [17]. Disease elimination as a public health problem, is defined by the WHO as a prevalence of ‘heavy-intensity infection of less than 1% [18]. Following these results, Mekele was declared “free of schistosomiasis” [18,19] and MDA campaigns were terminated for all schoolchildren in the city.

The objective of this study was to evaluate the sustainability of NALAs’ intervention, five years after project completion. Specific objectives included the identification and characterization of geographic areas prone to high rates of infection persistence, factors associated with control or resurgence of the disease, and the association between the behavioral change program and disease prevalence.

## 2. Methods and Materials

A cross-sectional study was conducted during November and December of 2018 in Mekele, Tigray. Mekele is made up of seven sub-cities, with a range of urban and semi-urban characteristics. Three main rivers cross the area: Elala River in the north, the northerly streaming Gereb Tsedo in the west, and Gereb Debri in the southern part of the city (supplemental material Figure S1). The overall school population in Mekele was 56,730, divided throughout 80 schools, at the time of the study.

Of the 38 schools that participated in the stool sample survey, 10 primary schools were selected to participate in the follow-up study in accordance with the following criteria: (i) historic data regarding prevalence of infections was available from the school; and (ii) the school was a public school within the boundaries of Mekele. Schools from both urban and semi-urban locations were included (semi-urban areas belong administratively to Mekele yet they are agricultural communities without commercial centers) (supplemental material, Table S1). An additional school (Debri School) was added to the study, to increase the representation of semi-urban schools located near rivers.

In accordance with WHO criteria for moderate and high-risk infected areas, schools were defined as persistent hot spots (PHS) if schistosomiasis prevalence was above 10% at the time of the study [20,21,22]. The number of students selected in each school was calculated with an equal representation of grades and gender. All participating students provided a stool sample for quantifying the presence of parasites and answered a knowledge, attitude and practice (KAP) questionnaire [23] to examine the impact and sustainability of the BCC program. The KAP questionnaire was developed by NALA (supplemental material, Appendix S1) for standardized use in monitoring and evaluation of all NALA’s programs in Ethiopia. The assistance of a trained Tigrinya translator was provided when necessary. Trained technicians examined the samples, detecting the presence of parasites using the wet mount technique [24]. Duplicate Kato–Katz [25] slides were prepared for each sample to be later examined in the laboratory of Tigray Health Research Institute (THRI). Eggs per gram (EPG) was used for the assessment, and the results were defined as light infection intensity (1 to 99 EPG), moderate infection intensity (100 to 399 EPG), and high infection intensity (400 or more EPG) [26].

### Statistical Analysis

Descriptive statistics were used to describe the study population by gender, age, and grade across all participating schools. Characteristics between students who were positive and negative for schistosomiasis were compared, alongside characteristics between schools with low prevalence of the parasite versus PHS schools using the chi-square test. Spearman’s correlation was used to examine the association between school location (proximity to rivers, urban setting vs. semi-urban setting) and disease prevalence, and to examine correlation between disease prevalence before intervention (2009) and at the time of the present study (2018). Logistic regression was used to examine predictors of both students and school characteristics for increased prevalence of infection. All analyses were performed with IBM SPSS statistics version 25.0. A *p*-value below 0.05 was considered significant.

## 3. Results

Six hundred and twenty-four participants were included in the study (Table 1). The mean age of the study population was 10.2 ± 2.3 years, with gender distribution similar between selected schools and representative of city-wide primary school gender distribution (49.5% females in our sample vs. 49.4% in the overall primary school population). Students from semi-urban areas were older than their counterparts in urban areas, 10.7 years vs. 9.8 years (*p* < 0.001).

Baseline STH prevalence was 8.1% in 2009, compared to post-intervention prevalence of 0.8% in 2018. Neither hookworms nor *Trichuris* were detected during the survey conducted in 2018. *Ascaris* was the sole STH identified, and was found in four of the 624 children enrolled in the study.

In 63.6% (7/11) of schools, prevalence of schistosomiasis remained as low as the prevalence measured five years earlier (less than 2%). These schools were defined as ‘responder’ schools. In the remaining schools, prevalence returned to pre-intervention levels and these schools were defined as PHS (4/11). Out of the four schools classified as PHS, two were classified as moderate risk areas with schistosomiasis prevalence of above 10% (Lachi and Debri), and two schools were classified as high risk areas, with a schistosomiasis prevalence of above 50% (Gembela and Feleg Daero).

A significant correlation (RR = 0.72, *p*-value = 0.03) was found between the prevalence of the infection in 2010 and that in 2018. All schools with a disease prevalence higher than 10% in 2010, (two years after initiation of the intervention), were all identified as PHS schools in 2018. Table S2 (supplemental material) describes prevalence of infection in 2010 and 2018 by school location (urban vs. semi urban) and distance from river (within or beyond 500 m).

Students from responder schools scored better on the KAP questionnaires [practice questions (4.2 ± 0.9 vs. 3.9 ± 0.9, *p*-value < 0.001) and attitude questions (4.6 ± 1.5 vs. 4.4 ± 1.4, *p*-value = 0.03)] compared to those from PHS schools. Table S3 (supplemental material) shows KAP results divided into the three KAP sections (knowledge, attitude and practice) by PHS schools and responder schools.

Gereb Tsedo and Adi Haki Schools had a zero to very low schistosomiasis rate in 2018. Feleg Daero School presented a disease prevalence of 70.6%. Students from Gereb Tsedo and Adi Haki Schools scored better in attitudes and practice questions in the KAP surveys compared to students from Feleg Daero School (*p*-values 0.041 and <0.001, respectively). A high prevalence of schistosomiasis (62%) infection was detected in Gembela School, located in urban surroundings and away from a river (>500 m). Children from this school (*n* = 21) attended lower grades (grade 3 vs. grade 4, *p*-value = 0.02) compared to their peers from the same age group from other schools. Additionally, scores on practice (3.8 ± 0.7 vs. 4.2 ± 0.9, *p*-value = 0.009) and knowledge questions (3.1 ± 1.0 vs. 4.0 ± 1.4, *p*-value = 0.002) were significantly lower compared to students from other urban schools. Table S4 (supplemental material) shows KAP results by school.

**Figure 1:**
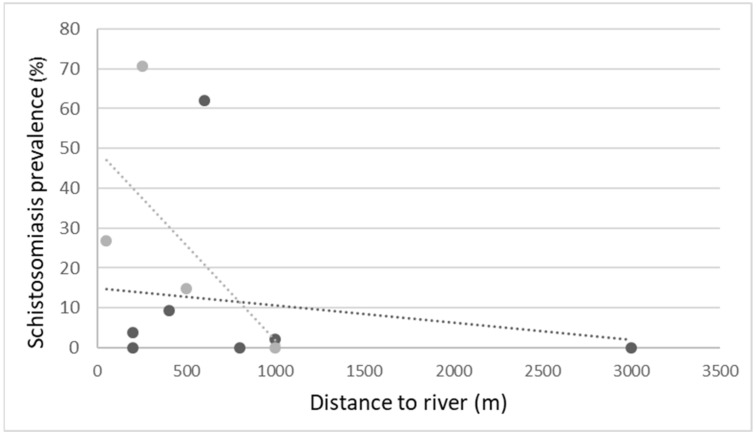
School distance from river and schistosomiasis prevalence, by location. Black dots: urban location; grey dots: semi-urban location.

Schistosomiasis prevalence positively correlated with age (positive samples: 10.7 ± 2.1 years vs. negative samples: 10.1 ± 2.3 years, *p*-value = 0.049), and boys were at higher risk of infection compared to girls (RR = 5.56, *p*-value < 0.001). Children living in semi-urban locations (*n* = 382) rather than urban ones (*n* = 242) had a significantly higher infection rate (22.7% vs. 5.5%, *p*-value < 0.001). Table S5 (supplemental material) shows study population demographics and infection prevalence by school. A negative correlation was found between the distance of schools from rivers and the infection rates in students, the magnitude of which varied between schools in semi-urban areas and schools in urban areas (all schools: RR = −0.60, *p*-value < 0.001; schools located in semi-urban areas: RR = −0.91, *p*-value = 0.001; schools located in urban areas: RR = −0.51, *p*-value = 0.001) (Figure 1).

## 4. Discussion

This study demonstrates the long-term success of NALA’s comprehensive intervention model for elimination of schistosomiasis. Five years’ post-intervention, low disease prevalence was sustained throughout the city, despite a cessation in FMoH delivered MDA. The sustained elimination achieved in the majority of schools was associated with increased knowledge, attitude, and practices of schoolchildren, attesting to the impact of the intervention.

Proximity of school to rivers was associated with increased disease prevalence, likely attributed to children and adults living close to rivers and using them for recreational and livelihood purposes [26]. However, three urban schools situated less than 500 m from a river were able to maintain low prevalence of infection. Two main differences were found between the responder and the non-responder schools that could account for these findings. Firstly, the two responder schools scored better in attitudes and practice questions in the KAP surveys. Whilst measuring the impact of a behavioral change intervention can present challenges [27], the KAP surveys were able to capture differences between schools. Second, Feleg Daero School is located in a semi-urban area while the two responder schools are located in urban areas. In contrast, Gembela School is located in an urban location, not adjunct to a river, yet had a relatively high prevalence of schistosomiasis. Children in Gembela School attended lower grades relative to peers their age in other schools. This might be an indicator of a lower socio-economic standard [28]. In addition, compared with the other urban schools, their KAP questionnaire scores were lower.

Infection rates increased with age, and boys showed higher infection rates compared to girls. These findings are in line with reports from other studies [3,29], including a longitudinal study conducted in central Sudan among children aged 6–15 [30], where boys had higher infection rates of schistosomiasis compared to girls. Similarly, in a cross-sectional study conducted in Gondar, Ethiopia [13], children above the age of 11 had higher infection rates compared to younger children. These rates are possibly a result of boys swimming naked as a daily activity, compared to girls who interact with water as they get older and have household responsibilities associated with the river. In a different study from south Ethiopia [31], schistosomiasis prevalence in girls was 7.7% and 17.1% in boys, and the most infected age group was that of 10–14 years old. While these factors might not be important in predicting PHS, they are essential when planning behavioral change activities since they enable targeted behavioral change interventions for specific at-risk populations.

A positive association was found between the prevalence of infection in the schools in 2010, two years into the intervention, and prevalence of infection in 2018. Schools with disease prevalence higher than 10% in 2010 were all defined as PHS schools in 2018. Schools with high infection rates following the deworming in 2010 were identified as PHS. As disease prevalence decreases, political and communal commitments to MDA and BCC interventions may decrease. The characteristics of a PHS must therefore be taken into account when planning, implementing, and phasing out interventions [20,21,22]. Within a single city, multiple sub-entities may exist, necessitating a more nuanced micro-mapping. This may permit ending an intervention in one area in favor of rigorous attention to other areas. In addition, MDA campaigns should be organized and conducted in a targeted manner. For example, population-based administration of drugs versus school-based administration may be advised in endemic areas. These areas will require greater attention in both conducting MDAs and continuing behavioral change support.

Our study has limitations. Records regarding infection intensity (eggs per gram stool-EPG) in the Kato–Katz testing were not available for the primary study, therefore we were not able to examine changes in infection intensity. Information regarding water and sanitation in the schools and communities and MDA campaigns was limited. Therefore, it was not possible to assess whether the findings were confounded by WASH improvements or infection treatment. Since a vector survey was not included in this study, it was not possible to assess the presence of infected snails as a possible confounder. Finally, a qualitative study amongst students, parents, school staff and other major stakeholders would have been useful in filling additional knowledge gaps [32]. Strengths of the study include the long-term follow-up NALA was able to perform in communities that received the initial intervention, while including diverse communities and geographical locations in a relatively large sample.

## 5. Conclusions

These findings validate the model implemented by NALA and their partners. They lend support to the importance of behavioral change and integrated, culturally sensitive interventions in achieving long lasting control of schistosomiasis in endemic areas. In addition, the study allowed us to identify both protective and risk factors associated with eradication and reinfection of schistosomiasis, respectively. These factors may assist in early detection of persistent hot spots and in the planning of future interventions.

## Figures and Tables

**Table 1:** Characteristics of study population by primary school location in Mekele, Ethiopia.

	Semi-Urban Schools		Urban Schools		Total *n* = 624	*p*-Value
	≤500 m from River *n* = 202	>500 m from River *n* = 40	≤500 m from River *n* = 165	>500 m from River		
Male	102 (50.5%)	20 (50%)	83 (50.3%)	110 50.7%)	315 (50.5%)	0.99
Female	100 (49.5%)	20 (50%)	82 (49.7%)	107 (49.3%)	309 (49.5%)	
Grade	4	4	3	4	4	0.015
Age, years	10.7 ± 2.5	10.9 ± 2.1	9.6 ± 2.0	10.1 ± 2.2	10.2 ± 2.3	<0.001

## Data Availability

Not applicable.

## Supplementary Materials

The following supporting information can be downloaded at: https://www.mdpi.com/article/10.3390/tropicalmed7090218/s1, Table S1: Participating primary schools in Mekele, Ethiopia; Table S2: Prevalence of infection by school location and distance from river; Table S3: KAP results (knowledge, attitude and practice) by PHS schools and responder schools; Table S4: KAP results (knowledge, attitude and practice) by school; Table S5: Study population demographics and infection prevalence, by school; Figure S1: Map of Mekele, the seven sub-divisions, main rivers, and the schools included in the study; Appendix S1. Knowledge Attitude Practice (KAP) survey developed by NALA 2019.
