# Supplementary material for: Sustainable Elimination of Schistosomiasis in Ethiopia—A Five-Year Follow-Up Study
Source: Trop Med Infect Dis. 2022 Sep 1;7(9):218. doi: 10.3390/tropicalmed7090218 (PMC9501490; doi:10.3390/tropicalmed7090218)
Supplement: Supplementary file 1 [file tropicalmed-07-00218-s001.zip › tropicalmed-1830511-supplementary.pdf]

Tropical Medicine and Infectious Disease

MDPI

Supplementary Materials: Sustainable Elimination of Schistosomiasis in Ethiopia—A Five-Year Follow-Up Study

Table S1. Participating primary schools in Mekele, Ethiopia.

Name of School	Location	Students Enrolled in Schools in 2018	Participating Students in 2018
Gereb Tsedo	urban	1436	56
Quiha	urban	1406	54
Ethio-China	urban	1253	48
Adi Haki	urban	1166	45
Ayder	urban	1660	64
Gembela	urban	550	21
Atse Yohannes	urban	2445	94
Aynalem	semi-urban	1044	40
Feleg Daero	semi-urban	774	34
Lachi	semi-urban	2863	108
Debri	semi-urban	1491	60
Total		16,088	624

Table S2. Prevalence of infection by school location and distance from river.

	Urban schools							Semi-urban schools					
	≤ 500m from river				> 500m from river			≤ 500m from river			> 500m		
	Gereb Tsedo	Adi Haki	Ayder	Gembela	Atse Yohannes	Ethio-China	Quiha	Debri	Feleg Daero	Lachi	Aynalem	n children in survey	n S.mansoni positive
S.mansoni 2010	0(0%)	1(3.3%)	ND	5(15.6%)	1(3.3%)	5(8.9%)	0(0%)	ND	19(44.2%)	6(5.5%)	1(%)	599	53(8.9%)
S.mansoni 2018	2(3.8%)	0(0%)	6(9.4%)	13(62%)	0(0%)	1(2.1%)	0(0%)	16(26.7%)	24(70.6%)	16(14.8%)	0(0%)	624	76(12.2%)

ND = No Data available.

Table S3. KAP results (knowledge, attitude and practice) by PHS schools and responder schools.

	Mean ± SD	Mean ± SD	Mean ± SD	
Knowledge (0–7 score)	4.00 ± 1.38	3.89 ± 1.37	3.96 ± 1.38	0.403
Attitudes (0–7 score)	4.65 ± 1.55	4.42 ± 1.42	4.57 ± 1.51	0.030
Practice (0–6 score)	4.19 ± 0.88	3.91 ± 0.99	4.09 ± 0.93	< 0.001
Total KAP score (0–20)	12.77 ± 2.75	12.21 ± 2.63	12.57 ± 2.72	0.003

Table S4. Median KAP results (knowledge, attitude and practice) by school.

School	Gereb Tsedo	Quiha	Ethio-China	Adi Haki	Feleg Daero	Gembela	Atse Yohann	Aynalem	Ayder	Lachi	Debri	Total	*p*-Value
	$n = 56$	$n = 54$	$n = 48$	$n = 45$	$n = 34$	$n = 21$	$n = 94$	$n = 40$	$n = 64$	$n = 108$	$n = 60$	$n = 624$	
Knowledge (0–7 score)	4	5	3	4	4	3	4	4	4	4	4	4	< 0.001
Attitudes (0–7 score)	5	5	5	5	4.5	5	5	4.5	4	5	4	5	< 0.001
Practice (0–6 score)	5	5	4.5	4	4	4	4	4	4	4	4	4	< 0.001
Total KAP score (0–20)	14	14	13	14	12.5	12	13	12.5	12.5	13	11	13	< 0.001

Table S5. Study population demographics and infection prevalence, by school.

	**Gereb Tsedo**	**Quiha**	**Ethio-China**	**Adi Haki**	**Feleg Daero**	**Gembela**	**Atse Yohann**	**Aynalem**	**Ayder**	**Lachi**	**Debri**	**Total**	***p*-Value**
	***n* = 56**	***n* = 54**	***n* = 48**	***n* = 45**	***n* = 34**	***n* = 21**	***n* = 94**	***n* = 40**	***n* = 64**	***n* = 108**	***n* = 60**	***n* = 624**	
**Sex (*n*, %)**													
Boys	29 (51.8%)	29 (53.7%)	25 (52.1%)	23 (51.1%)	18 (52.9%)	10 (47.6%)	46 (48.9%)	20 (50%)	31 (48.4%)	53 (49.1%)	31 (51.7%)	315 (50.5%)	0.99
Girls	27 (48.2%)	25 (46.3%)	23 (47.9%)	22 (48.9%)	16 (47.1%)	11 (52.4%)	48 (51.1%)	20 (50%)	33 (51.6%)	55 (50.9%)	29 (48.3%)	309 (49.5%)	
Grade (median)	3.5	4.5	3.5	3	3	3	4	4	3	4.5	4	4	< 0.001
Age, years	9.9 ± 2.3	10.4 ± 2.2	9.5 ± 1.8	9.4 ± 1.9	10.1 ± 2.2	9.5 ± 1.4	10.3 ± 2.5	10.9 ± 2.1	9.5 ± 1.7	10.6 ± 2.3	11.2 ± 2.7	10.2 ± 2.3	< 0.001

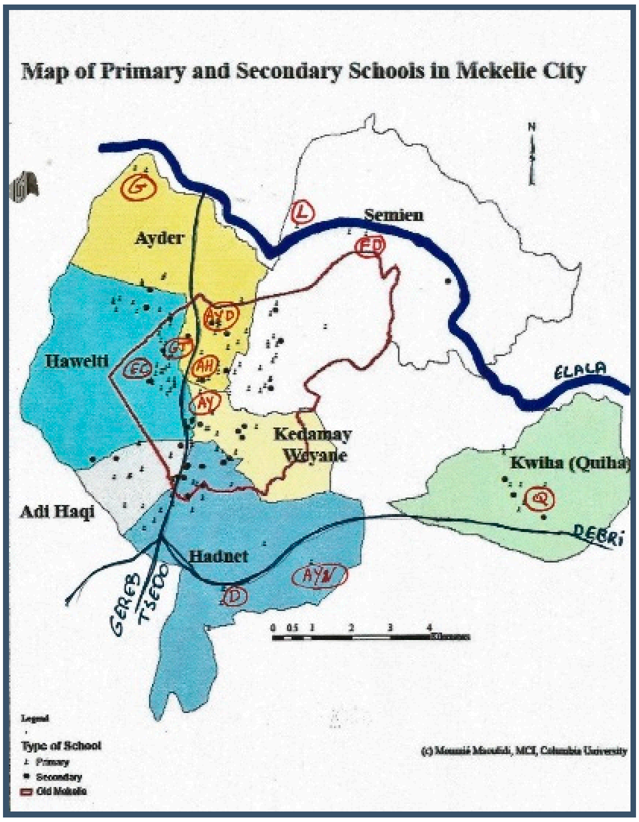

Figure S1. Map of Mekele City, the seven sub-divisions, main rivers, and the schools included in the study. G = Gembela school; L = Lachi school; FD = Feleg Daero school; Q = Quiha school; AYD = Ayder school; GT = Gereb Tsedo school; EC = Ethio-China school; AH = Adi Haki school; AY = Atse Yohannes school; AYN = Aynalem school; D = Debri school.

Appendix S1. Knowledge Attitude Practice (KAP) survey developed by NALA 2019

KAP (Knowledge Attitude Practice) survey Date ………………

Name of School…………………………… number…………..

Age ………….. Sex ………….. Grade …………..
Address ……………………….

Part A- Knowledge. Mark one answer.

1. Have you heard of intestinal worm infections?

a. Yes

b. No

2. Can a person get infected with intestinal worms by walking barefoot?

a. Yes

b. No

3. Can a person be infected with intestinal worms from a mosquito bite?

a. Yes

b. No

4. Which of the following is a symptom of intestinal worms?

a. Red, itchy eyes

b. Diarrhea

c. Fever

5. How does trachoma (eye infection) spread from a sick person to a healthy person?

a. Sharing face cloths

b. Swimming in water

c. Reading at night

6. To prevent infection when eating vegetables, a person needs to….

a. Do nothing

b. Wash, cook, or peel the vegetables

c. Put them in the sun

7. Can open defecation cause disease?

a. No

b. Yes

B. Attitudes

8. When medication for disease is given in school, I feel comfortable taking the medication.

a. Agree

b. Disagree

9. Wearing shoes is important for my health.

a. Agree

b. Disagree

10. Intestinal parasites is a big health problem in my school.

a. Agree

b. Disagree

11. It is okay if children defecate in the water or the field.

a. Agree

b. Disagree

12. It is better to use a latrine.

a. Agree

b. Disagree

13. I feel embarrassed if my friends see me open defecate.

a. Agree

b. Disagree

C. Practice.

Mark one answer

14. I swim/play/wash myself in the river…

a. Never

b. Sometimes

c. Everyday

15. If a latrine is available, I use it….

a. Never

b. Sometimes

c. All the time

16. I wear shoes….

a. Never

b. Sometimes

c. All the time

<u>Mark all correct answers</u>

17. I wash my hands when…

a. When they look or feel dirty

b. Before eating or preparing food

c. After defecation

d. Never

18. When I clean my hands I….

a. Wipe on a cloth

b. Use water only

c. Use water and ash

d. Use water and soil

e. Use water and soap

10. I wash my face…..

a. When it is dirty

b. When there is discharge from the eyes or nose

c. One time a day

d. One time a week

e. Never
